# Integrating Bulk RNA and Single-cell transcriptome to explore the role of glycan-related genes in lung adenocarcinoma

**DOI:** 10.7150/jca.115989

**Published:** 2025-07-24

**Authors:** Keyun Zhu, Changqing Yang, Guixin Wang, XingKai Wang, Chengbin Lin, Yingxi Li, Ningning He, Yao Tian, Weiyu Shen

**Affiliations:** 1 Department of Thoracic Surgery, The Affiliated LiHuiLi Hospital of Ningbo University, Ningbo, Zhejiang, 315040, China.; 2 Department of Respiratory and Critical Care Medicine, Tianjin Medical University General Hospital, Tianjin, 300052, China.; 3 The First Department of Breast Cancer, Key Laboratory of Cancer Prevention and Therapy, Tianjin's Clinical Research Center for Cancer, National Clinical Research Center for Cancer, Key Laboratory of Breast Cancer Prevention and Therapy, Tianjin Medical University Cancer Institute and Hospital, Tianjin Medical University, Tianjin, 300060, China.; 4 Immunology Department, Key Laboratory of Immune Microenvironment and Disease (Ministry of Education), Tianjin Medical University, Tianjin, 300070, China.

**Keywords:** glycan, glycosylation, immune regulation, lung adenocarcinoma, prognosis, treatment

## Abstract

**Background:** Despite significant advancements in the diagnosis and therapeutic management of lung adenocarcinoma (LUAD), patient outcomes continue to be suboptimal, primarily attributable to the intricate of the tumor microenvironment (TME). Recently, attention has been paid to the role of glycans and their glycosylation modifications in tumor progression.

**Methods:** In the present investigation, we performed analyses to identify 13 glycan-related genes with prognostic significance in LUAD. High- and low-risk groups were distinguished by a constructed model of glycan-related genes. Single-cell analysis was performed to investigate the TME in LUAD. Drug screening analysis was utilized to predict potential candidate drugs.

**Results:** High-risk patients exhibited aggressive tumor progression. Further single-cell analysis revealed that tumor cells expressing high-risk glycan-related genes displayed enhanced interactions with immune and stromal cells, suggesting that aberrant glycosylation and glycan biosynthesis may contribute to worse outcomes in LUAD by promoting immune suppression. Furthermore, based on the molecular characteristics, we identified several potential candidate drugs for personalized treatment, including docetaxel, alpelisib, and gefitinib.

**Conclusion:** Our study found that glycan-related genes could alter the composition of immune cell infiltration in LUAD tumor tissues and might affect the interaction between immune cells and tumor cells through intercellular section signals, resulting in the inability of immune cells to normally initiate immune responses against tumor cells. These findings offer new biological perspectives of glycan-related genes in shaping the TME and potential targets for personalized LUAD treatment.

## Introduction

Lung adenocarcinoma (LUAD) imposes a substantial global disease burden 1-3. Despite demonstrated efficacy of various therapeutic approaches, the clinical outcomes for advanced or metastatic LUAD remain suboptimal due to treatment non-specificity and acquired drug resistance 4-6. To advance precision medicine in oncology, contemporary research has increasingly adopted integrated multi-omics strategies to systematically characterize both cell-autonomous oncogenic mechanisms and heterotypic cellular interactions within the tumor microenvironment (TME) 7, 8. Consequently, unraveling the molecular mechanisms of LUAD, particularly those related to the TME, has become a focal point in cancer immunotherapy research.

Glycans, as glycosylation modifications of cell surface and secreted proteins, contribute to tumor development and progression 9, 10. Glycosylation regulates interactions within TME, which relates to tumor migration and immune evasion 11, 12. Malformed glycan structures and dysregulated glycosylation pathways on tumor cell surfaces have been implicated as key mediators of tumor immune evasion 13. Studies in ovarian cancer suggest that tumor cells may form a glycoprotein barrier composed of abnormal glycans, thereby preventing immune cell recognition 14. Additionally, glycosylation alterations can modulate immune checkpoint interactions, particularly within the PD-1/PD-L1 axis, ultimately influencing immunotherapy efficacy 15. Given these findings, targeting glycans and dysregulated glycosylation represents a promising strategy in cancer treatment 16, 17. However, the specific role of glycan-related genes and their possibility for individual treatment in LUAD remain key challenges to be addressed.

Among the study, 13 glycan-related genes were identified with prognostic significance to develop a glycan-related model to stratify LUAD patients based on prognosis. Integrated multi-omics analysis revealed that tumor progression in the high-risk cohort may be driven by augmented intercellular communication networks within the tumor microenvironment. Finally, based on differential activation of glycan-related pathways, we predicted potential candidate drugs for personalized treatment. Our investigation elucidates novel mechanisms by which glycan-related genes modulate tumor cell biology and tumor microenvironment interactions to promote LUAD progression, while simultaneously establishing their dual significance as prognostic biomarkers and therapeutic targets.

## Materials and Methods

### Data sources and analysis platforms

Transcripts per kilobase million (TPM) transcriptomic data were scaled by log2 transform and obtained from UCSC Xena 18. Corresponding clinical data, including tumor stage, survival status, and gender, were extracted from cBioPortal. Glycan-related genes were selected from glycan biosynthesis pathways and extracted from the kyoto encyclopedia of genes and genomes (KEGG) collection in the molecular signatures database (MSigDB). Single-cell transcriptomic dataset was obtained from GSE189357 dataset on gene expression omnibus (GEO), excluding *in situ* carcinoma samples to focus exclusively on invasive LUAD 19. Candidate drugs were screened from the genomics of drug sensitivity in cancer (GDSC) 20 and connectivity map (CMap) databases. R 4.4.1 was utilized for data analysis.

### Gene screening and model construction

A number of 76 glycan-related genes were intersected with upregulated genes in LUAD. The prognostic significance of these genes was estimate by cox regression. The relationships among the 13 prognostic glycan-related genes were evaluated by spearman correlation. A Cox regression model was then developed to predict LUAD prognosis based on these 13 genes, with gene weights rounded to two decimal places. The performance of the model for the first-, third-, and fifth-year survival was assessed using nomogram. The discrimination ability was assessed by area under the curve (AUC) based on receiver operating characteristic (ROC) curves. Calibration curves were used to evaluate the accuracy of the model. Clinical benefit was estimated by decision curve analysis (DCA). ROC curves were generated using “timeROC” package (version 0.4), while the nomogram and calibration curves were conducted by “rms” package (version 7.0-0) with 500 bootstrap resampling iterations. DCA curves were performed by “rmda” package (version 1.6). Patients were stratified into high- and low-risk groups based on the median glycan-related risk score derived from the model.

### Differential genes and functional analyses

Differential gene expression analyses were carried out in LUAD versus normal samples, and the high- versus low-risk glycan-related gene expression groups. The “limma” package (version 3.60.4) was used for differential analysis 21, with a standard of log2 fold change > 0 and P-value < 0.05. Differentially expressed genes (DEGs) were conducted for functional analysis by “clusterProfiler” package (version 4.10.1) 22. Reference datasets for gene functions, pathways, and cellular processes were obtained from MSigDB. Gene set enrichment analysis (GSEA) deemed pathways as significant with the criteria of P-value < 0.05 23. Immune cell infiltration levels were estimated using CIBERSORTx, with immune cell reference datasets sourced from CIBERSORTx and previous studies 24. Group comparisons of immune infiltration scores were conducted using Wilcoxon tests and P-value < 0.05 was regarded as difference.

### Single-cell data processing and analysis

Single-cell data were processed under the pipeline of Seurat package (version 4.4.0). Cells were incorporated only if they met the quality control criteria: nFeature > 200, nCount > 500, mitochondrial gene proportion < 20%. Cells failing to meet these criteria were eliminated. Besides, genes expressed in fewer than three cells will be excluded from subsequent analysis. Batch effects were removed using the Harmony package (version 1.2.0) 25, and doublet removal was performed with DoubletFinder (version 2.0.3) 26. SCTransform was applied to identify variable genes. Cell annotation was conducted based on specific cell markers defined in previous studies 19. For annotated epithelial cells, inferCNV was used to identify tumor cells. According to the median AUC score, high- and low-glycan-expression tumor cells clusters were grouped 27, computed using the AUCell package (version 1.26.0) with the 13 glycan-related genes as the reference set. To explore their interactions within TME, inter-cellular crosstalk was evaluated by CellChat package (version 1.6.1) 28, focusing on secreted signaling pathways to assess interaction strength between different cell types.

### Drug screening and prediction

Drug screening was conducted to identify potential compounds for LUAD patients in different groups. Drug sensitivity predictions were generated by oncoPredict package 29 basing on bulk RNA data. IC50 values were obtained from the GDSC database. To identify candidate therapeutic drugs, the top 150 up- and down-regulated genes were selected among DEGs for drug prediction in the CMap database.

## Results

### Identification of glycan-related genes with prognostic value

**Figure 1:**
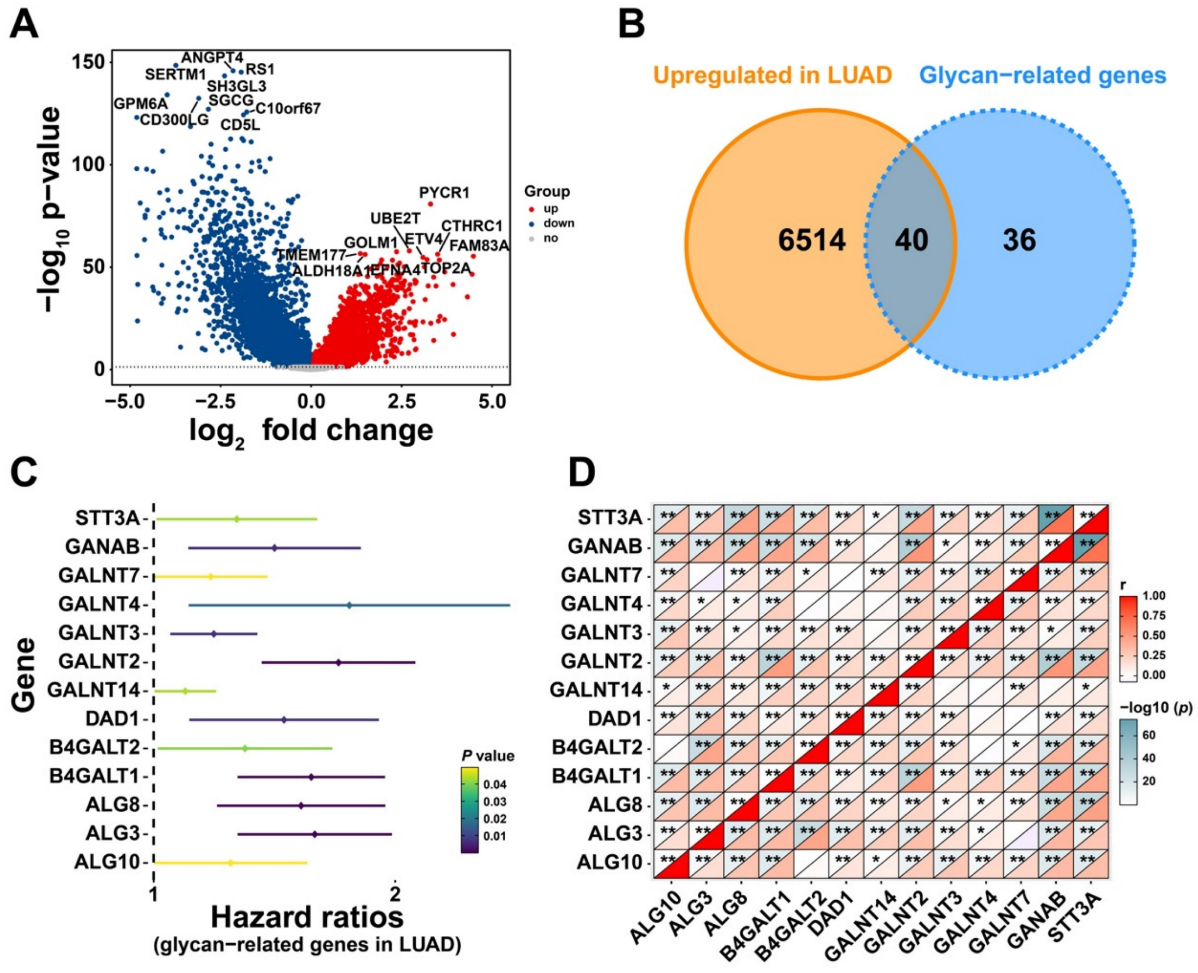
** Identification of Glycosylation-Related Genes with Prognostic Value. (A)** DEGs between LUAD and normal patients. **(B)** Upregulated glycan-related genes in LUAD. **(C)** Prognostic glycan-related genes in LUAD. **(D)** Correlation plot between prognostic glycan-related genes in LUAD.

To explore the effect of glycan-related genes in LUAD, a differential expression analysis was performed between LUAD and normal samples. The results revealed that genes such as PYCR1 and UBE2T were significantly upregulated in LUAD patients (Figure 1A). Among these upregulated differentially expressed genes, 40 genes were associated with glycan synthesis (Figure 1B). This analysis identified glycan-related genes exhibiting abnormal expression in LUAD, suggesting their potential involvement in disease progression. To further evaluate their clinical relevance, we assessed their prognostic significance and identified 13 glycan-related genes with prognostic value (Figure 1C). Correlation analysis demonstrated a strong positive correlation among these genes (Figure 1D), implying that they may exert synergistic effects and play a critical role in LUAD development.

### Construction of a glycan-related risk model

**Figure 2:**
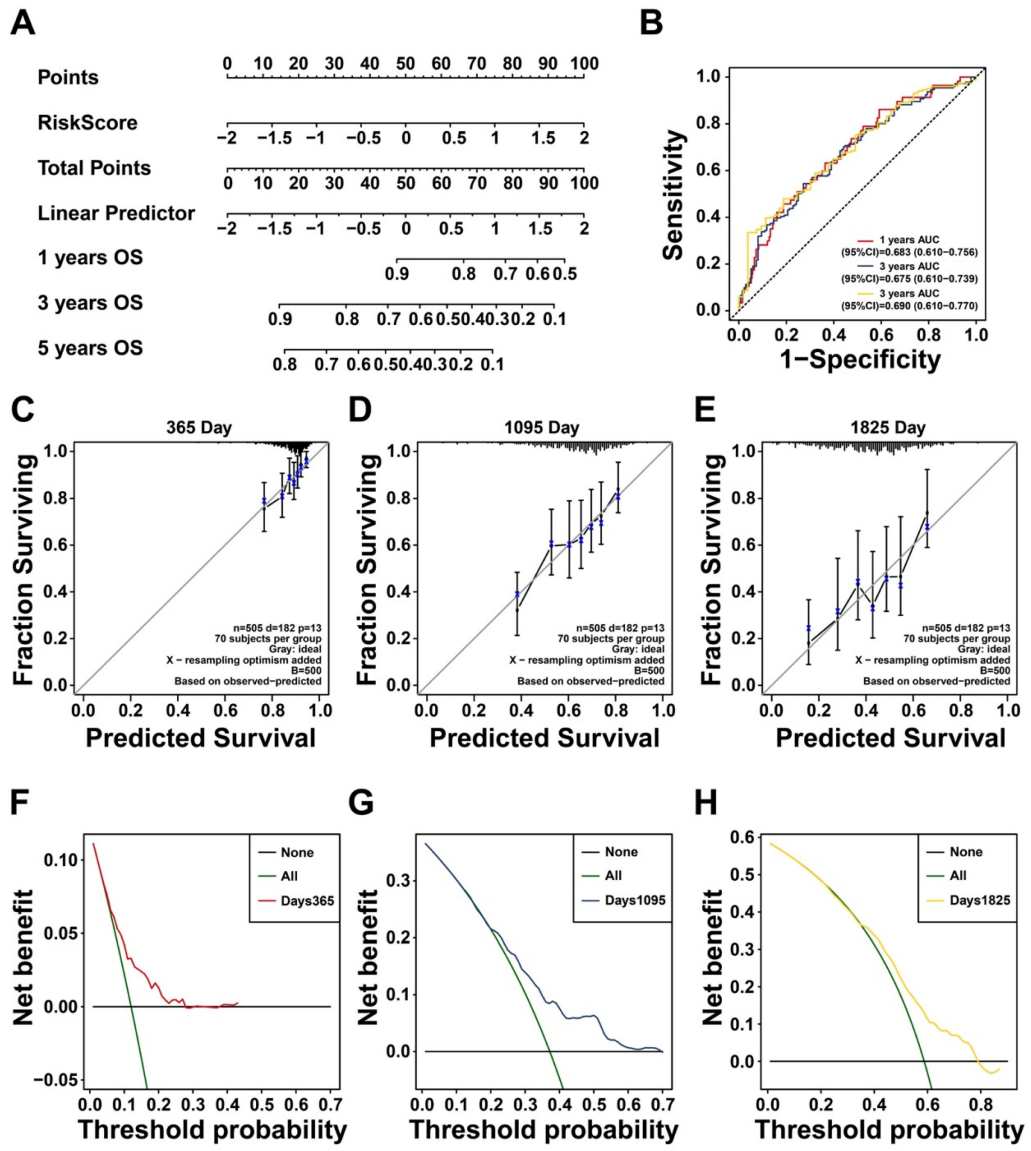
** Construction of a Prognostic Model Based on Prognostic Glycan-Related Genes. (A)** Nomogram of glycan-related prognostic model. **(B)** ROC curve of 1-, 3-, and 5- years prognosis of the model. **(C-E)** Calibration curve of (C) 1-, (D) 3-, and (E) 5- years prognosis of the model. **(F-H)** DCA curve of (F) 1-, (G) 3-, and (H) 5- years prognosis of the model.

Given the prognostic significance of these 13 glycan-related genes, a glycan-related model was developed to predict LUAD patients' prognosis. The model formula is presented as follows: risk score = 0.33114*GALNT2 + 0.219025*B4GALT1 + 0.220311*ALG3 + 0.290613*ALG8 + 0.068599*GANAB + 0.098837*GALNT3 + 0.203968*DAD1 + 0.084303*GALNT4 - 0.07864*B4GALT2 - 0.33933*STT3A - 0.002*GALNT14 + 0.065194*GALNT7 - 0.06925*ALG10. Based on patient gene expression levels, individual risk scores were calculated to predict survival probability at specific time points (Figure 2A). The AUC for the first-, third-, and fifth-year survival remained above 0.65 (Figure 2B), indicating good predictive performance. Calibration curves for the first-, third-, and fifth-years further confirmed the accuracy and robustness in predicting LUAD survival outcomes (Figure 2C-E). Furthermore, decision curve analysis (DCA) revealed that the proposed model offered significant clinical net benefit across a wide threshold probability range while optimizing healthcare resource utilization (Figure 2F-H). Collectively, this prognostic model effectively stratifies LUAD patients and showed the possible effect of these genes in LUAD progression.

### Biological characteristics affected by glycan-related genes

**Figure 3:**
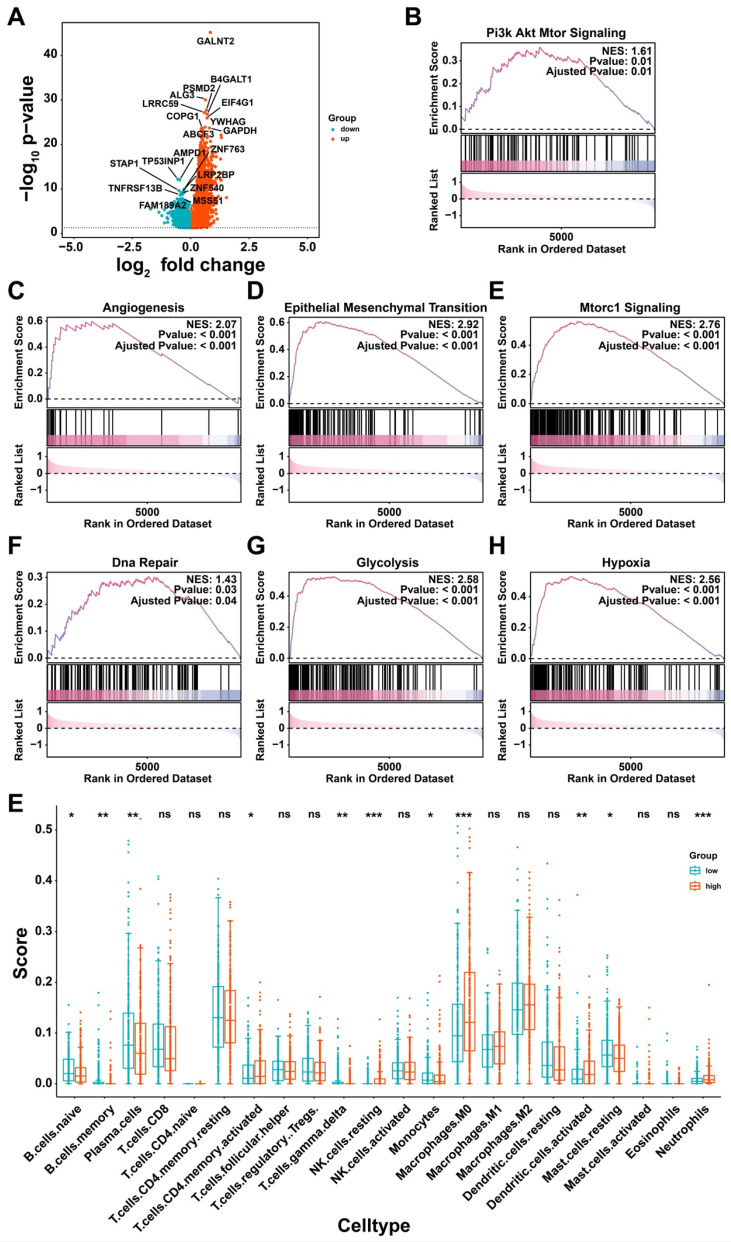
** Potential biological process affected by glycan-related genes. (A)** DEGs between high- and low-risk group in LUAD. **(B-H)** GSEA revealing pathways in high- versus low-risk group in LUAD. (B) Pi3k Akt Mtor Signaling. (C) Angiogenesis. (D) Epithelial Mesenchymal Transition. (E) Mtorc1 Signaling. (F) Dna Repair. (G) Glycolysis. (H) Hypoxia. (I) Immune cell infiltration between high- and low-risk group in LUAD.

To elucidate how glycan-related genes have impact on LUAD, high- and low-risk groups were divided based on the prognostic model. Significant differences in tumor staging were observed between two groups, suggesting that the high-risk group may be characterized as tumor progression (Table S1). Differential expression analysis identified additional genes, including upregulated PSMD2, EIF4G1, and COPG1 in high-risk group (Figure 3A). It turned out that PI3K/Akt, angiogenesis, and epithelial-mesenchymal transition (EMT) pathways were activated in high-risk group (Figure 3B-D). Furthermore, mTORC1 signaling, DNA repair, glycolysis, and hypoxia response pathways were also activated (Figure 3E-H), suggesting that glycan-related genes may promote tumor progression via multiple cellular mechanisms. Immune cell infiltration analysis revealed distinct immune profiles between the two groups (Figure 3E). Notably, M0 macrophages and activated dendritic cells (DCs) were more prevalent in high-risk group, but γδ T cells owned abundance infiltration in low-risk group. Taken together, glycan-related genes may contribute to LUAD progression and poor prognosis by modulating key oncogenic pathways and altering immune cell infiltration patterns.

### Role of glycan-related genes in tumor cells

**Figure 4:**
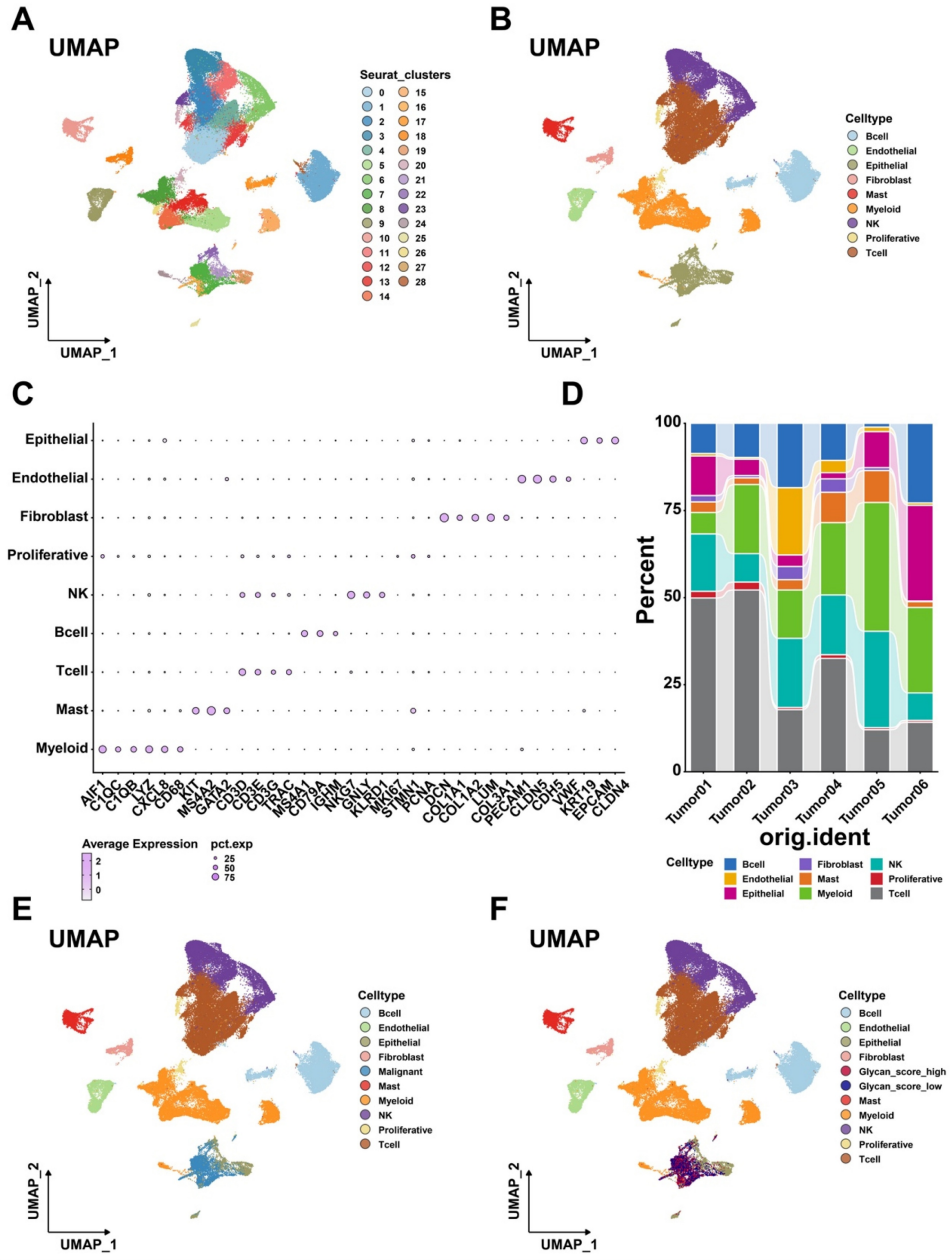
** Single cell transcriptome analysis. (A)** Different cell clusters in Uniform Manifold Approximation and Projection (UMAP). **(B)** Nine cell types in LUAD. **(C)** Annotation markers of each cell types in LUAD. **(D)** Proportion of each cell types in different samples. **(E)** Exhibition of each cell types including malignant cells in UMAP. **(F)** Exhibition of each cell types including glycan score high and low cells in UMAP.

To further investigate the role of glycan-related genes in tumor cells, single-cell RNA sequencing analysis was conducted 30. After quality control and dimensionality reduction, 72,412 cells were clustered into 29 groups (Figure 4A), which were subsequently annotated into nine distinct cell types (Figure 4B). High expression of specific cell markers validated the accuracy of these annotations (Figure 4C). The distribution of these cell populations varied across samples (Figure 4D), reflecting TME heterogeneity in LUAD patients. Copy number variation (CNV) analysis identified tumor cells among epithelial cells (Figure S1, Figure 4E). To examine glycan-related gene expression in tumor cells, we stratified them into high- and low-glycan score groups (Figure 4F).

**Figure 5:**
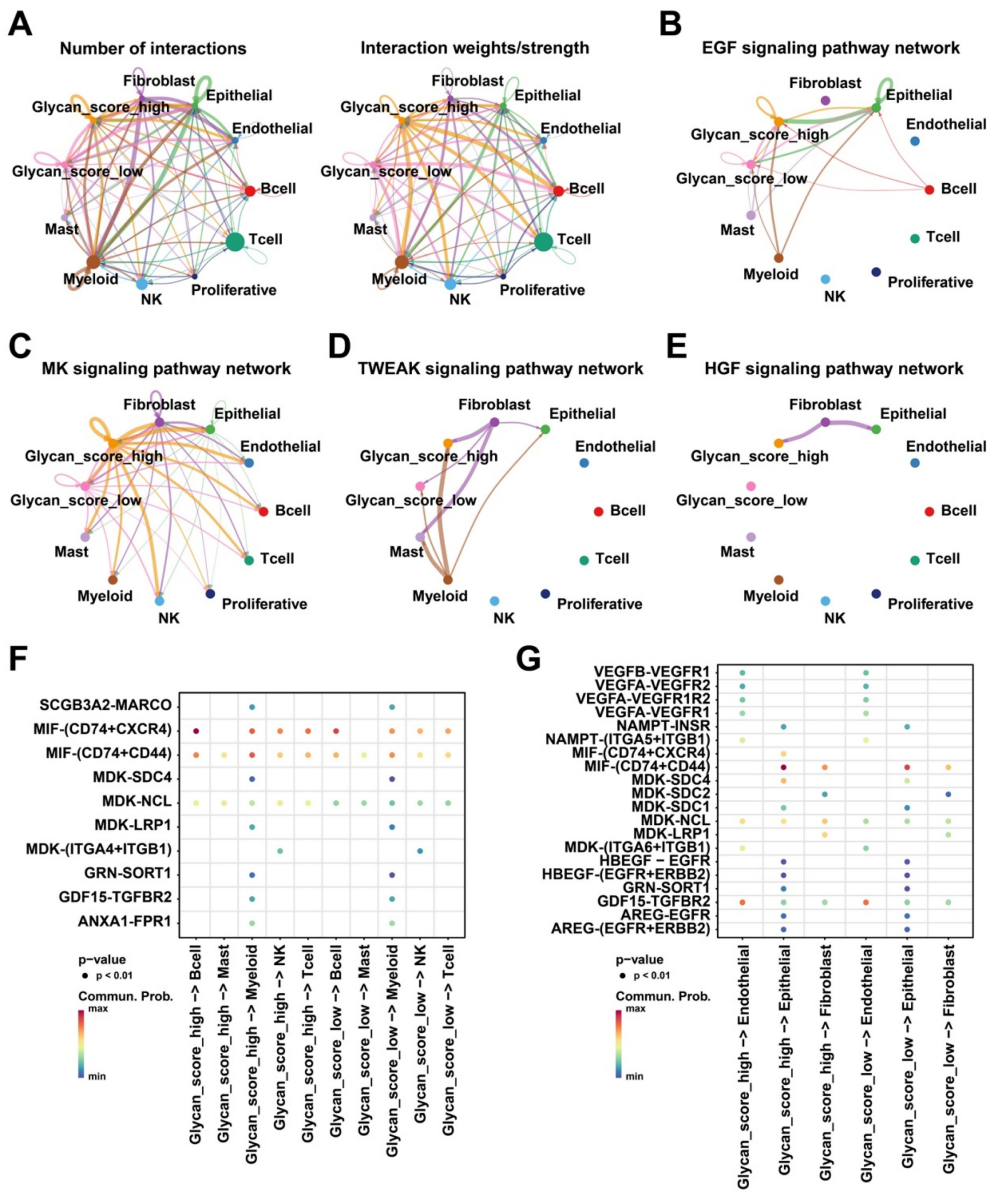
** Intercellular communication within TME. (A)** Numbers and strength of interactions within TME. **(B)** EGF, **(C)** MK, **(D)** TWEAK, and **(E)** HGF signaling pathway networks within TME. **(F)** Intercellular communication between glycan-related malignant cells and immune cells. **(G)** Intercellular communication between glycan-related malignant cells and stromal cells.

Tumor cells influence disease progression through interactions with other cells in TME 31. Intercellular communication analysis uncovered extensive cell-cell crosstalk networks within the LUAD tumor microenvironment, exhibiting significant heterogeneity in interaction strength. (Figure 5A). Although the number of interactions did not differ between the two glycan score groups, tumor cells with higher glycan scores exhibited stronger interactions with other cell types. Notably, the EGF, MK, and TWEAK signaling pathways were more actively engaged in high-glycan score tumor cells than in low-glycan score cells (Figure 5B-D). Additionally, the HGF signaling pathway was exclusively observed in the high-glycan score group (Figure 5E). Receptor-ligand analysis further confirmed that tumor cells with high glycan scores exhibited stronger interactions with immune and stromal cells, particularly through MIF and MDK ligands (Figure 5F-G). These findings suggest that glycan-related genes enhance intercellular communication, potentially facilitating LUAD progression.

### Prediction of potential drugs targeting glycan-related genes

**Figure 6:**
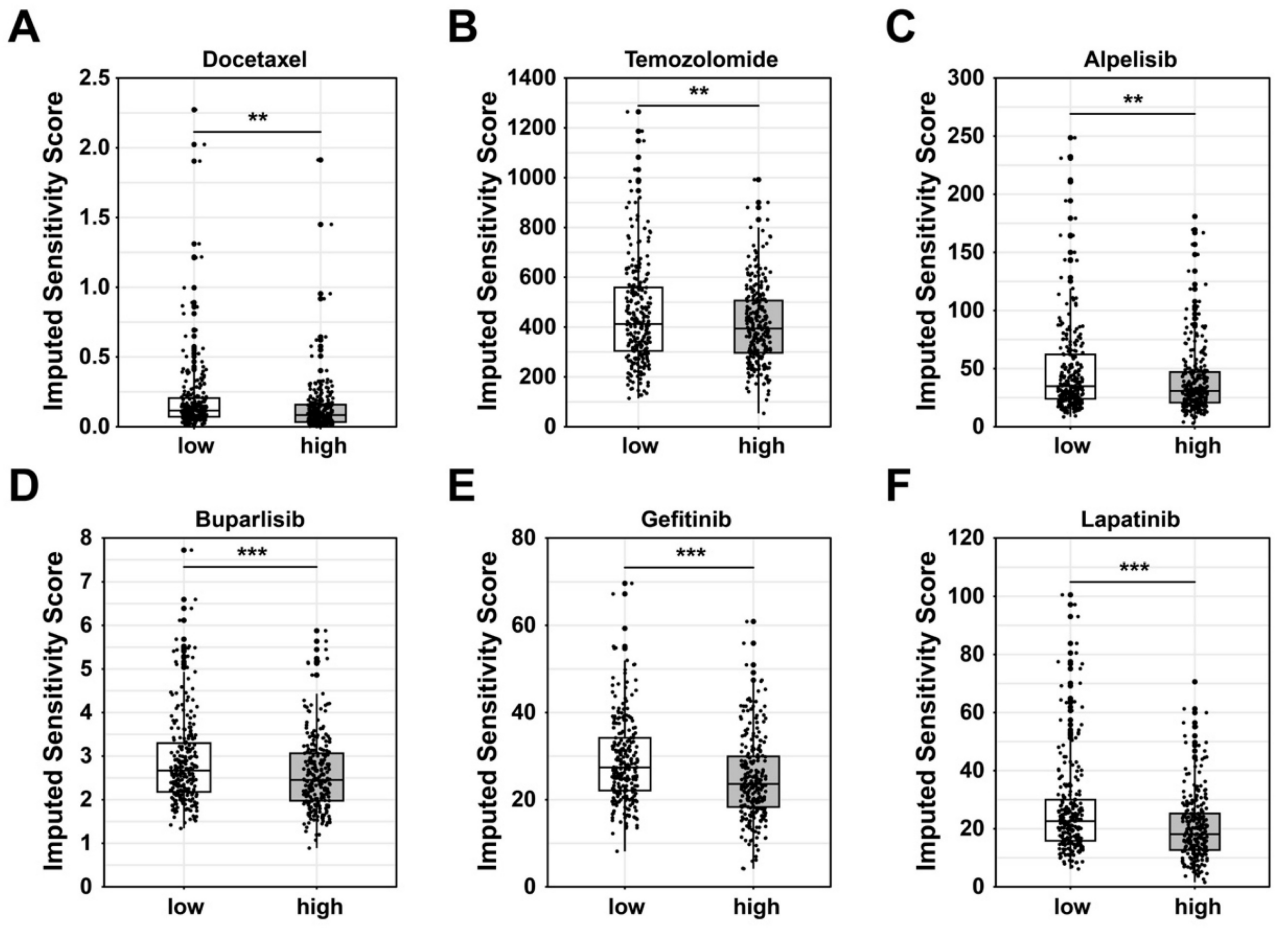
** Potential drugs between high- and low-risk group in LUAD.** Sensitivity score of **(A)** Docetaxel, **(B)** Temozolomide, **(C)** Alpelisib, **(D)** Buparlisib, **(E)** Gefitinib, and **(F)** Lapatinib.

**Figure 7:**
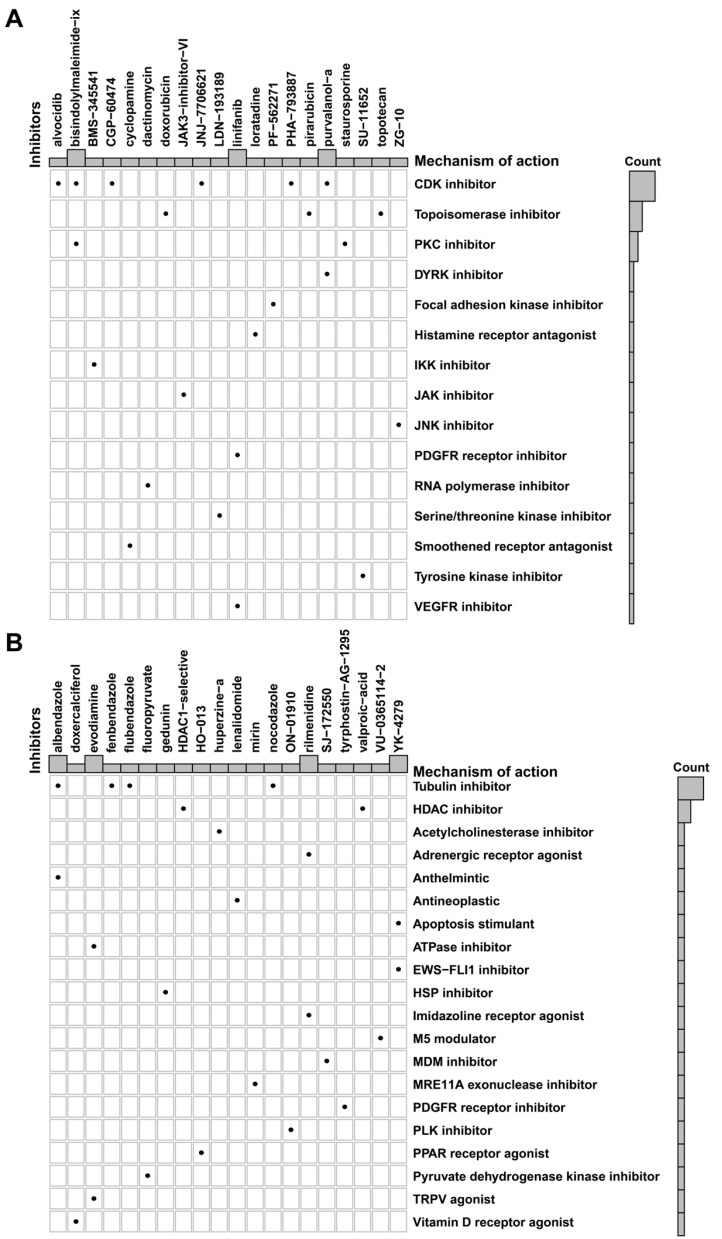
** Potential small molecular drugs in high- and low-risk group in LUAD.** Potential small molecular drugs in **(A)** high-risk group and **(B)** low-risk group.

Based on the putative oncogenic properties of glycan-related genes in LUAD, we systematically screened for potential therapeutic compounds stratified by high- and low-risk patient subgroups. The sensitivity was evaluated among widely used cancer drugs. Docetaxel and temozolomide owned higher sensitivity in high-risk group (Figure 6A-B). Interestingly, patients in high-risk group were more sensitive to PI3K inhibitors, alpelisib and buparlisib (Figure 6C-D). Similarly, EGFR inhibitors such as gefitinib and lapatinib might be more effective in high-risk patients (Figure 6E-F). Furthermore, we investigated novel small-molecule compounds with limited prior application in oncological contexts. For example, SU11652 was identified as a potential therapeutic agent for high-risk group, as well as lenalidomide for low-risk group (Figure 7A-B). Thus, glycan-related gene might be therapeutic strategies for LUAD patients, offering opportunities for personalized treatment approaches.

## Discussion

Glycosylation, a critical structural modification to form glycan, exists in various biological processes 32. Aberrant glycosylation has been implicated in tumor progression 30 and may influence tumor cells by modulating the tumor immune microenvironment and facilitating immune evasion 33. Several studies have focused on the function of glycosylation and glycans in LUAD 34, 35. In this study, we identified prognostic glycan-related genes and developed a predictive model for LUAD patient survival. Through multi-omics analyses, we demonstrate that glycan-related genes contribute to tumor progression by promoting tumor growth and modulating the TME. Moreover, these candidate genes represent promising targets for the development of novel therapeutic interventions. Our findings provide a foundation for stratifying LUAD patients based on glycan-related gene expression and offer insights into personalized diagnosis and treatment strategies.

A number of 13 glycan-related genes were screened as prognostic markers in LUAD. Consistent with previous studies, these genes have been shown to promote tumor growth, migration, therapy resistance, and other factors contributing to poor patient prognosis 36-38. For example, in colorectal cancer, GALNT2 modifies the O-glycosylation of the AXL receptor tyrosine kinase, thereby regulating AXL levels and promoting tumor invasion 39. ALG3 can induce glycosylation of TGF-β receptor II (TGFBR2), thereby conferring resistance to conventional therapies 40. Additionally, DAD1, a well-established apoptosis regulator in tumors 41, is also a part of the oligosaccharyltransferase (OST) located on the endoplasmic reticulum 17. Alterations in OST subunits can lead to protein misfolding in the endoplasmic reticulum, thereby contributing to oncogenesis 42. Upon incorporating these genes into a cox proportional hazards model, the predictive performance for LUAD patient survival was robust. Unlike previous LUAD prognostic models, which primarily examined the roles of individual genes, our study provides comprehensive analyses to reveal the involvement of glycan-related genes in tumor progression 43, 44. This expands our understanding of LUAD pathogenesis by investigating glycan synthesis and modification. Primarily, this study highlights the regulatory role of glycan-related genes in LUAD tumorigenesis through systematic characterization of their dysregulated glycosylation profiles. Besides, the model demonstrates robust discriminative capacity, diagnostic accuracy, and clinical applicability, validating its reliability. Most importantly, our results position glycan-related pathways as crucial determinants of LUAD prognosis, providing novel avenues for both mechanistic investigation and therapeutic development.

Given the ability of the prognostic model to effectively differentiate LUAD patients into two groups, biological processes associated with tumor progression were significantly activated in high-risk group. Notably, the PI3K/Akt, angiogenesis, and EMT pathways have been widely reported to drive tumor progression 45-47. Interestingly, previous studies have suggested that abnormal glucose metabolism in tumors induces metabolic reprogramming in tumor cells 48, 49. The mTOR pathway, which is closely linked to cellular nutrient status 50, shows a crucial effect in metabolic reprogramming. In tumors, mTOR activation facilitates metabolic reprogramming by enhancing glycolysis while suppressing mitochondrial oxidative phosphorylation 51, 52. Under hypoxic conditions, glycolysis-driven lactic acid production by tumor cells acidifies the TME, thereby fostering an immunosuppressive environment 53. Additionally, tumor cells can enhance DNA repair mechanisms and stabilize immune checkpoint proteins, leading to chemotherapy resistance and immune evasion 54. These findings suggest a potential interplay between metabolic reprogramming in LUAD. Furthermore, differences in immune cell infiltration were observed between the high- and low-risk groups. M0 macrophages, despite lacking full polarization, retain the ability to promote tumor cell proliferation, migration, and invasion, making them linked to tumor progression 55. In contrast, antigen presentation relies on activated DCs, which contributes to T cell activation in the TME 56. Whereas no significant distinction was detected in CD8+ T cell infiltration, potentially due to abnormal glycosylation and glycan modifications on tumor cell membranes in high-risk group, which may impair the anti-tumor activity of dendritic cells 17, 57, 58. Additionally, γδ T cells, which exhibit anti-tumor properties and are associated with favorable prognoses in various cancers 59, 60, showed reduced infiltration in high-risk groups. This demonstrated that glycan-related alterations might contribute to an immunosuppressive TME. Collectively, these findings indicate that abnormal glycosylation driven by glycan-related genes may activate pro-tumor pathways, reprogram tumor metabolism, and create an immune evasive microenvironment, ultimately leading to disease progression and poor prognosis in LUAD patients.

According to bulk RNA-seq data, single-cell transcriptomic analysis was performed to delineate the precise roles of glycosylation-related aberrations in malignant cells and their crosstalk with the TME. Our analysis demonstrated that malignant cells can be effectively stratified according to distinct glycan-related gene expression patterns. Notably, tumor cells with high glycan scores exhibited stronger signaling interactions within the TME and actively communicated with immune and stromal cells via multiple pathways. Consistent with previous findings, abnormal glycosylation and glycan synthesis have a profound impact on the TME 61, potentially influencing ligand-receptor interactions, thereby modulating immune and stromal cell behavior and contributing to disease progression and poor treatment outcomes 30, 62. Our study also identified enhanced EGF, MK, TWEAK, and HGF signaling between high-glycan-score tumor cells and others. N-glycosylation in the Golgi complex generates ligands for lectins, including the EGF receptor (EGFR) 63, suggesting that aberrant glycosylation may activate the EGF pathway in tumor cells. Additionally, MET, a receptor for HGF, contains N-glycosylation modification sites, and its interaction with EMT enhances tumor treatment resistance 64, 65. Furthermore, MK growth factor and TWEAK contribute to tumorigenesis, angiogenesis, and EMT via the PI3K/Akt and Fn14 signaling pathways, respectively 66, 67. In tumor-immune cell crosstalk, macrophage migration inhibitory factor (MIF) was identified as a pivotal signaling molecule. Notably, MIF as an immunosuppressive factor in the TME, promotes tumor progression via an immune evasive environment 68. Similar to other components of TME, abnormal glycosylation and glycan synthesis can alter glycoprotein structures on immune cell surfaces, enabling tumor immune evasion 69, 70. Therefore, the single-cell analysis further corroborates that glycosylation associated alterations play a pivotal role in LUAD progression and immune tolerance development.

Given the established involvement of glycosylation and glycan biosynthesis in LUAD progression, we conducted computational drug sensitivity predictions to identify potential therapeutic agents. Among chemotherapeutic agents currently used in clinical settings, docetaxel and temozolomide demonstrated greater sensitivity in the high-risk group. These drugs exert cytotoxic effects by inhibiting mitosis and inducing tumor cell death via DNA alkylation 71. As previously discussed, high-risk LUAD tumors exhibit abnormal glycolysis-induced DNA repair mechanisms. As a DNA alkylating agent, temozolomide may exhibit enhanced efficacy against these dysregulated DNA repair mechanisms, positioning it as a viable therapeutic candidate for high-risk LUAD patients. Furthermore, PI3K pathway activation, a hallmark of the high-risk group, suggests that PI3K inhibitors such as alpelisib and buparlisib may provide therapeutic benefits 72, 73. Similarly, EGF pathway activation was also observed in this group, consistent with findings that abnormal glycosylation and glycan synthesis can activate EGF signaling. This aligns with the mechanisms of gefitinib and lapatinib, which are EGFR-targeting agents and may exhibit enhanced efficacy in high-risk patients 74, 75. These findings manifest that patients in high-risk group might respond favorably to these targeted therapies, highlighting potential treatment strategies based on glycosylation associated alterations. To expand therapeutic options and explore personalized treatment strategies, we also performed small-molecule drug predictions based on DEGs. It identified SU11652 and lenalidomide as a promising candidate for high- and low-risk group respectively. SU11652, a tyrosine kinase inhibitor (TKI), may effectively counteract downstream oncogenic signaling triggered by abnormal glycosylation-mediated receptor activation 76, 77. In contrast, lenalidomide, an anti-tumor agent with immunomodulatory properties, has shown potential efficacy in treating solid tumors, making it a viable therapeutic option for low-risk LUAD patients 78. Overall, these potential drugs offer promising candidates for future personalized treatment targeting aberrant glycosylation and glycan biosynthesis in LUAD.

Overall, the main finding is that glycan-related genes may lead to abnormal interactions between tumor cells and immune cells by influencing glycan synthesis and abnormal glycosylation in tumor cells. This abnormal intercellular interaction may promote the formation of the immunosuppressive tumor microenvironment and thereby lead to poor tumor treatment response and tumor progression. Most current studies have suggested that the type of glycan and the glycosylated molecular structure on the cell membrane in lung cancer patients are different from those in healthy controls 34. For instance, studies have indicated that small extracellular vesicles with different glycosylated molecules may appear in the peripheral blood of lung cancer patients, and the content of glycans in the saliva of lung cancer patients increases with abnormal N-glycan molecules 79, 80. These studies indicate that for the detection of glycan molecules, such as through mass spectrometry analysis, early screening of lung cancer patients can be achieved through convenient liquid biopsy. Unlike these studies, our research focuses on glycan synthesis genes and discovers that abnormal expression of genes may also alter the biological behavior of tumor cells by influencing glycan molecules and glycosylation. More importantly, our research further discovered that glycan-related genes may affect the interaction between tumor cells and immune cells, forming a complex tumor immune microenvironment and hindering the therapeutic effect of patients with LUAD. Therefore, we screened potential drugs that could target this process and identified potentially suitable drugs for future treatment.

As a secondary analysis of publicly available datasets, our study has several inherent limitations that should be acknowledged. The UCSC Xena database only provides clinical information related to LUAD. However, if patients have diseases that affect prognosis or the expression of glycan related genes, it may lead to an overestimation of the impact of glycan-related genes on the prognosis of patients with LUAD. Thus, the identified glycan-related genes and the prognostic model require validation in larger, independent patient cohorts to confirm their clinical relevance. While this glycan-related prognostic model offers insight into LUAD progression, its predictive accuracy must be compared to existing models. Further research is needed to explore integrative models that combine glycan-related biomarkers with other molecular features to enhance prognostic precision. Our study provides speculative insights into pathways, metabolic programs, and cellular interactions associated with LUAD. However, the specific molecular mechanisms underlying these processes remain unvalidated and require experimental confirmation. The therapeutic efficacy of the potential drugs must be validated through experiments and clinical trials to determine their safety and clinical benefits. Though there were several limitations, valuable insights were presented to show the role of glycosylation and glycan synthesis in LUAD progression.

## Conclusion

The effect of glycan-related genes was investigated in LUAD, highlighting their involvement in prognostic assessment, tumor development, and the formation of an immunosuppressive TME. Based on these findings, potential therapeutic agents for LUAD patients are identified. This research offers valuable insights into personalized management and treatment strategies targeting abnormal glycosylation and glycan synthesis in LUAD.

## Supplementary Material

Supplementary figure and table.

## Abbreviations

LUAD: lung adenocarcinoma
TCGA: The Cancer Genome Atlas Program
TPM: Transcripts per kilobase million
GEO: Gene expression omnibus
DEGs: differentially expressed genes
log2FC: log2 fold-change
KEGG: Kyoto Encyclopedia of Genes and Genomes
GSEA: Gene Set Enrichment Analysis
CNV: copy number variation
GDSC: Genomics of Drug Sensitivity in Cancer
CMap: Connectivity map
AUC: Area under the curve
ROC: Receiver operating characteristic
DCA: Decision curve analysis
EMT: epithelial-mesenchymal transition
DC: dendritic cells
TME: tumor microenvironment
TGF-β: transforming growth factor-β
TGFBR2: TGF-β receptor II
OST: Oligosaccharyltransferase
EGFR: epidermal growth factor receptor
TKI: Tyrosine kinase inhibitor
