# Supplementary material for: Integrating Bulk RNA and Single-cell transcriptome to explore the role of glycan-related genes in lung adenocarcinoma
Source: J Cancer. 2025 Jul 24;16(11):3314–28. doi: 10.7150/jca.115989 (PMC12374833; doi:10.7150/jca.115989)
Supplement: Supplementary file 1 — Supplementary figure and table. [file jcav16p3314s1.pdf]

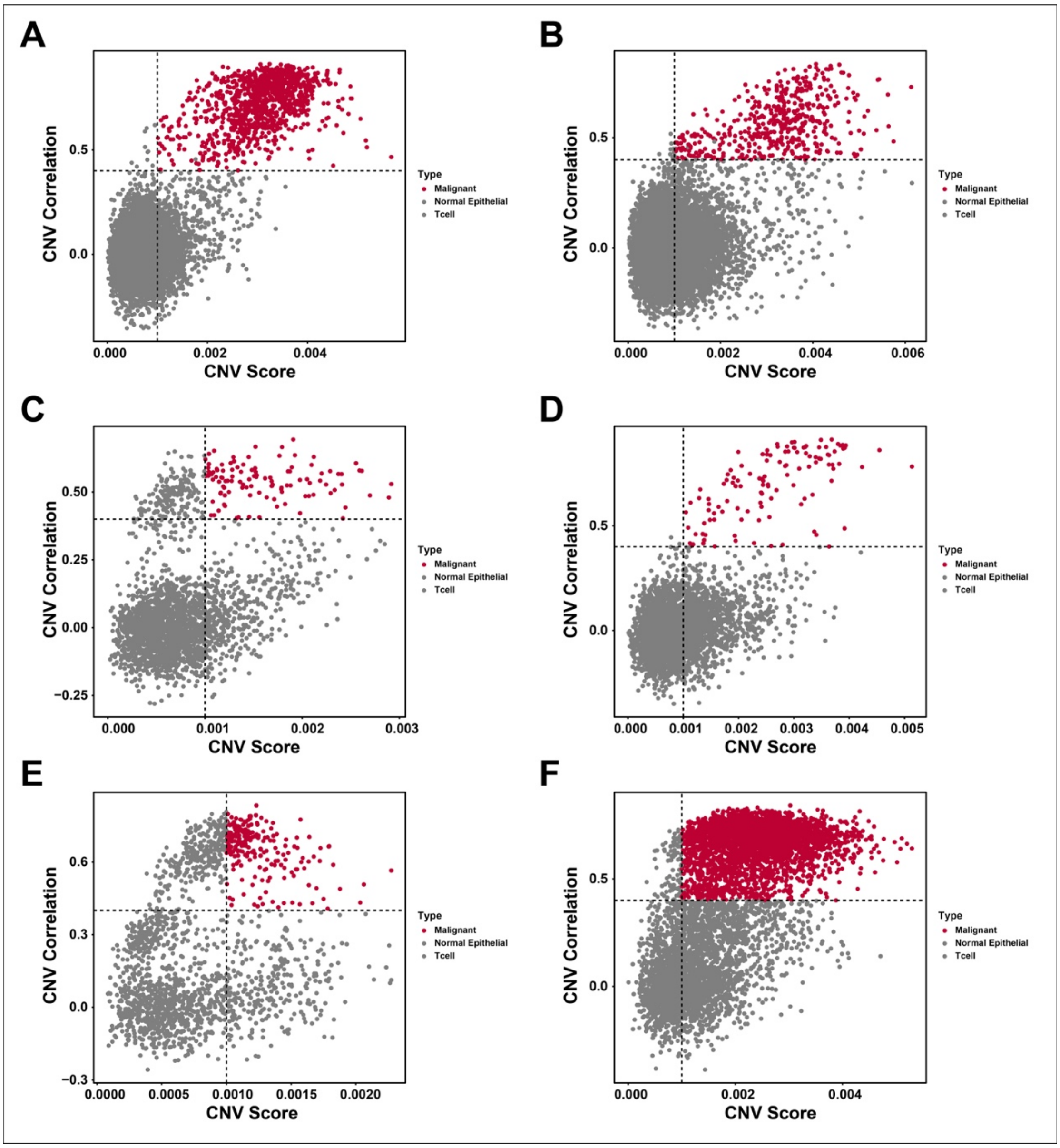

Figure S1. CNV analyses of different samples. Dot plot of CNV score and CNV correlation for malignant, normal epithelial and T cells from (A) tumor01 to (F) tumor06

Table S1 Clinical features between high- and low-risk group

Variables	Total (n = 505)	high (n = 253)	low (n = 252)	p
M_stage, n (%)				0.641
M0	336 (67)	173 (68)	163 (65)	
M1	17 (3)	11 (4)	6 (2)	
M1a	2 (0)	1 (0)	1 (0)	
M1b	5 (1)	2 (1)	3 (1)	
MX	141 (28)	64 (25)	77 (31)	
NA	4 (1)	2 (1)	2 (1)	
N_stage, n (%)				< 0.001
N0	328 (65)	142 (56)	186 (74)	
N1	95 (19)	58 (23)	37 (15)	
N2	69 (14)	48 (19)	21 (8)	
N3	2 (0)	1 (0)	1 (0)	
NA	1 (0)	1 (0)	0 (0)	
NX	10 (2)	3 (1)	7 (3)	
Stage, n (%)				0.002

NA	8 (2)	4 (2)	4 (2)	
Stage I	272 (54)	116 (46)	156 (62)	
Stage II	120 (24)	66 (26)	54 (21)	
Stage III	80 (16)	53 (21)	27 (11)	
Stage IV	25 (5)	14 (6)	11 (4)	
T_stage, n (%)				0.081
T1	169 (33)	72 (28)	97 (38)	
T2	270 (53)	142 (56)	128 (51)	
T3	45 (9)	26 (10)	19 (8)	
T4	18 (4)	12 (5)	6 (2)	
TX	3 (1)	1 (0)	2 (1)	
Sex, n (%)				0.967
Female	272 (54)	137 (54)	135 (54)	
Male	233 (46)	116 (46)	117 (46)	
